# Valuable Phytochemicals: Extracts and Extraction Techniques, Analyte Isolation, and Bioactive and Nutritional Properties

**DOI:** 10.3390/plants15050810

**Published:** 2026-03-06

**Authors:** Fábio Junior Moreira Novaes, Francisco Radler de Aquino Neto

**Affiliations:** 1 Departamento de Química, Universidade Federal de Viçosa, Avenida Peter Henry Rolfs, s/n, Campus Universitário, Viçosa 36570-900, MG, Brazil; 2 Laboratório de Apoio ao Desenvolvimento Tecnológico (LADETEC/IQ-UFRJ), Instituto de Química, Universidade Federal do Rio de Janeiro, Rio de Janeiro 21941-598, RJ, Brazil

Phytochemicals are secondary metabolites synthesized by plants to mediate environmental interactions and ensure their survival [1,2,3]. Within plants, they perform vital functions such as (i) defense, acting as phytoalexins against pathogens and deterrents against herbivores; (ii) attraction, by providing color, aroma, or flavor to recruit pollinators; and (iii) signaling, as growth hormones and in response to abiotic and biotic stresses [1,2,3,4,5]. Regarding human health, while these compounds are not classified as macronutrients required for immediate survival, they play a crucial role in long-term physiological optimization due to their protective and disease-preventive properties, which have garnered significant scientific and industrial interest [4,5].

The articles in this Special Issue address phytochemicals and bioactive extracts, focusing on expanding molecular knowledge on plants and evaluating strategies for phytochemical extraction, isolation, and chemical characterization, as well as their application after processing. Considering the commercial value of these metabolites, these studies prioritize approaches focused on optimizing extraction yield, reducing costs, and mitigating impacts on human health and the environment. In this context, aspects related to the dynamics of phytochemicals throughout the plant life cycle, variations in their levels during maturation, their potential as biomarkers, and comparisons between conventional methods and emerging green technologies were investigated. Additionally, targeted analytical and omics approaches, combined with experimental design, were employed to improve the efficiency and sustainability of these processes.

## 1. Contributions

Arsenault et al. [6] evaluated the delta-9-tetrahydrocannabinol (THC) and total cannabidiol (CBD) contents in 14 *Cannabis sativa* cultivars to verify sampling strategies and legal compliance with the maximum THC limit (0.3% *w*/*w*) [7]. The plants were grown between 2020 and 2022, sampled weekly, dried, extracted with methanol, and analyzed via GC-FID. The results showed that all cultivars presented a high risk of exceeding the legal limit at full maturity, with THC levels increasing from August to September and then stabilizing or decreasing at the end of October, possibly due to climatic conditions. Furthermore, significant individual variability was observed among plants of the same cultivar, demonstrating that sampling only one or two plants is not representative of larger batches, contradicting legal guidelines [8]. Therefore, it is necessary to consider the phytochemical variability of cultivars in order to develop more accurate sampling strategies. In conclusion, this study highlights that comprehensive characterization of chemical profiles and maturation stages is essential to drive the development of improved cultivars. Such knowledge enables the production of plants with standardized phytochemical compositions, ensuring compliance with agro-industrial objectives and stringent regulatory frameworks.

Antonio et al. [9] performed chemical characterization of *Ocotea delicata* Vicent using an untargeted metabolomics approach and UHPLC-HRMS, aiming to overcome the limitations of purely morphological identification and expand phytochemical knowledge of the species. The study identified 44 metabolites, including quinic acid and its derivatives, aporphine, and benzylisoquinoline alkaloids, as well as flavonoids, filling a relevant chemical gap and expanding the inventory of Brazilian biodiversity. The authors demonstrated that the metabolomic profile can act as a chemical fingerprint that is useful for chemotaxonomic studies, although analysis of a larger number of samples is needed for validation. Applying metabolomics to phytochemical studies opens the way for identifying new bioactive compounds, authenticating species and extracts, performing quality control, studying chemical variability, and supporting conservation strategies, sustainable use, and pharmaceutical and agro-industrial applications.

De Faria et al. [10] proposed, developed, and validated Direct Hot Solid–Liquid Extraction (DH-SLE) as an innovative green chemistry method for extracting lipids from coffee beans. The study employed a 3^3^ Full Factorial Design to optimize the sample/solvent ratio, temperature, and time, directly comparing the results with those from the Soxhlet method, a standard recommended by the AOAC [11]. DH-SLE achieved extraction yields statistically equivalent to those of Soxhlet extraction, without any alteration in fatty acid composition, as verified by GC-MS. However, it far surpassed the classic method by drastically reducing the sample, solvent, and energy consumption, as well as requiring a shorter extraction time and eliminating water consumption. The method allows for multiple simultaneous extractions, greater analytical productivity, and a 98.79% reduction in cost per sample, positioning it as a highly efficient, safe, and environmentally responsible alternative. DH-SLE can be explored in the identification, characterization, and quality control of phytochemicals—especially in the efficient extraction of bioactive compounds—and can also be integrated into omics approaches and sustainable analytical protocols, expanding its use in research and the natural products industry.

Lee et al. [12] identified the need for safer natural alternatives to conventional antibiotics in the treatment of skin infections caused by *Staphylococcus aureus* and, in this context, analyzed polyphenols, especially flavonoids and anthraquinones, extracted from *Cassia alata* (Linnaeus) Roxburgh. The study evaluated the use of *C. alata* leaves for treating atopic dermatitis, comparing three aqueous extraction systems based on methanol. Characterization by HPLC-DAD identified four valuable polyphenols—aloe-emodin, astragalin, kaempferol, and rhein—with greater recovery in the 75% *v*/*v* methanol/water extract, which also showed potent antioxidant and antimicrobial activity against *S. aureus* and promoted healing of human keratinocytes without cytotoxicity. The results highlight the importance of choosing the right extraction technique to maximize recovery of bioactive compounds and reinforce the potential of phytochemicals for the development of safe herbal medicines, cosmeceuticals, and natural products. In light of the results presented, it is understood that one of the first steps in the development of procedures is optimizing the composition of the extraction solvent mixture, as well as the extraction and isolation methods, with the aim of maximizing biological activity. This approach is crucial to consolidate the recovery of valuable phytochemicals and their application in efficient and safe therapeutic formulations.

Xiao et al. [13] investigated borneol essential oil (BEO), a byproduct of the steam distillation of *Cinnamomum camphora*, with the aim of evaluating its antibacterial activity against *Staphylococcus epidermidis* and its anti-inflammatory effects through exploring the mechanisms of action and the synergistic potential of its constituents. To this end, they performed broth microdilution tests to determine the minimum inhibitory concentration (MIC), mechanistic assays of bacterial cell wall disruption and macromolecule leakage, and anti-inflammatory evaluations in LPS-stimulated RAW 264.7 macrophages. Network pharmacology was employed to identify key bioactive compounds and modulated inflammatory pathways. The results showed that, although BEO and pure borneol had the same MIC, BEO exhibited superior antibacterial effects, demonstrating multicomponent synergism, and reduced the production of TNF-α, IL-1β, and IL-6 in a dose-dependent manner. The network pharmacology analysis indicated that borneol is primarily responsible for these effects, with β-caryophyllene, limonene, and camphor contributing by acting on multiple inflammatory and metabolic pathways. In conclusion, BEO emerges as a multi-target therapeutic agent with antibacterial and immunomodulatory relevance. From a phytochemical perspective, the study reinforces the value of whole extracts, the importance of isolating and characterizing bioactive analytes, and the value of exploring co-products as sustainable sources of valuable phytochemicals for multifunctional therapeutic formulations.

Manzo et al. [14] investigated essential oils from native Patagonian plants to identify effective, selective, and sustainable organic alternatives for controlling *Varroa destructor*, given the challenges posed by the use of synthetic acaricides, such as parasite resistance, environmental impacts, and adverse effects on *Apis mellifera*. To this end, they carried out chemical characterization of essential oils of *Adesmia boronioides*, *Dysphania multifida*, and *Senecio filaginoides* and conducted biological assays to evaluate acaricidal activity, toxicity in adult bees and larvae, and repellent effects. The results demonstrated that, although all of the oils showed activity against *V. destructor*, *A. boronioides* and *D. multifida* showed significant toxicity against bees, while *S. filaginoides*, which is rich in α-pinene, exhibited high acaricidal efficacy, high selectivity, an absence of adverse effects, and a residual effect. These findings indicate that *S. filaginoides* is a promising candidate for the development of environmentally safe and economically viable natural products. From a phytochemical perspective, the study highlights the value of volatile metabolites, especially monoterpenes, as valuable phytochemicals, reinforcing the importance of isolating bioactive analytes, investigating synergisms, and optimizing green extraction techniques, thus expanding possibilities for sustainable applications in beekeeping and pollinator protection within agroecological systems.

## 2. Final Synthesis: Knowledge Gaps and Future Research

**Figure 1:**
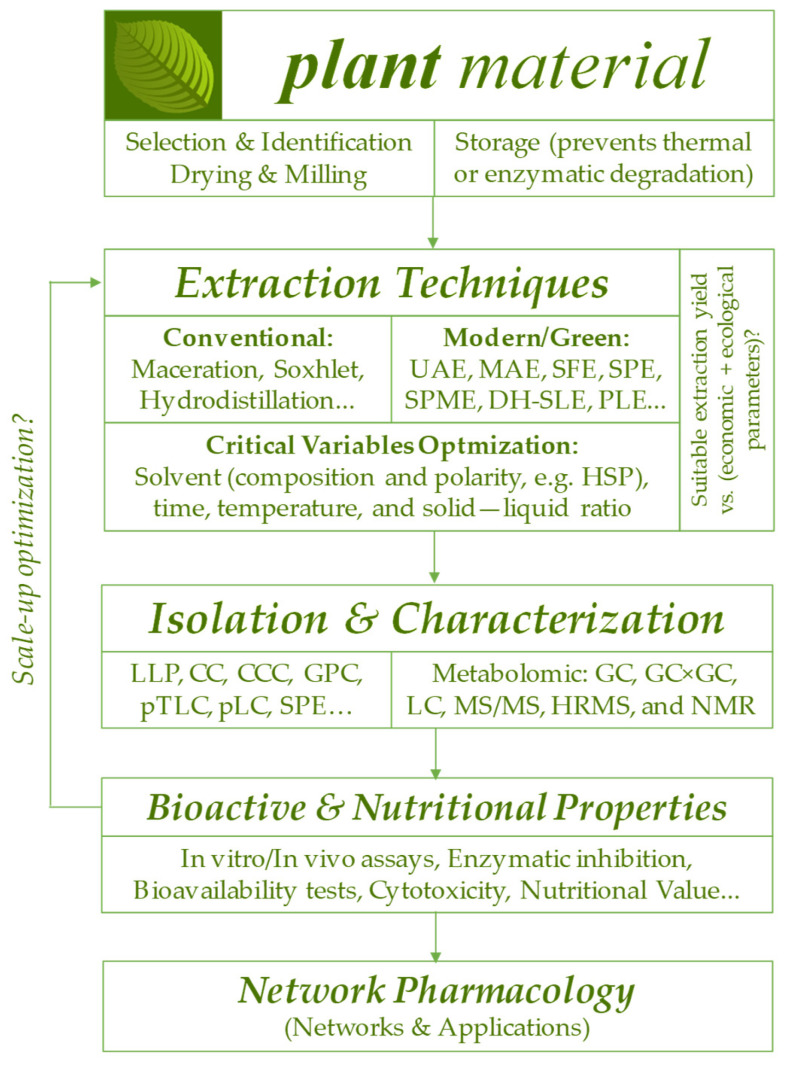
Methodological roadmap for phytochemical research.

Many plant species have known traditional uses but lack molecular validation and extraction standardization that would enable their sustainable and safe entry into the global market. One approach to filling this gap involves transforming raw botanical knowledge into actionable chemical and biological data, which can be achieved by integrating modern emerging technologies such as metabolomics, network pharmacology, and green techniques for phytochemical extraction and isolation. To assist researchers in navigating this complexity, Figure 1 provides a systematic methodological roadmap, illustrating the essential stages from biomass preparation to bioactivity validation. Consequently, the focus shifts toward sustainable industrial scaling-up of these processes and investigation of the economic viability of replacing synthetics with these phytochemicals on a large scale, as well as ensuring that the exploitation of these species (especially those from tropical forests or remote regions) does not lead to extinction, maintaining a commitment to biodiversity and responsible profitability, which opens the door for many future research projects on the topic of this Special Issue, “*Valuable Phytochemicals: Extracts and Extraction Techniques, Analyte Isolation, and Bioactive and Nutritional Properties*”.

## Data Availability

No new data were created or analyzed in this study. Data sharing does not apply to this article.

## Abbreviations

The following abbreviations are used in this manuscript:

THC	Delta-9-Tetrahydrocannabinol
CBD	Total Cannabidiol
GC	Gas Chromatography
FID	Flame Ionization Detector
UHPLC	Ultra-High-Performance Liquid Chromatography
HRMS	High-Resolution Mass Spectrometry
DH-SLE	Direct Hot Solid–Liquid Extraction
AOAC	Association of Official Analytical Chemists
GC-MS	Gas Chromatography–Mass Spectrometry
HPLC	High-Performance Liquid Chromatography
DAD	Diode Array Detection
BEO	Borneol Essential Oil
MIC	Minimum Inhibitory Concentration
TNF-α	Tumor Necrosis Factor Alpha
IL-1β	Interleukin 1 beta
IL-6	Interleukin 6
UAE	Ultrasound-Assisted Extraction
MAE	Microwave-Assisted Extraction
SFE	Supercritical Fluid Extraction
SPE	Solid Phase Extraction
SPME	Solid Phase Microextraction
PLE	Pressurized Liquid (or Accelerated Solvent) Extraction
HSP	Hansen Solubility Parameters (an in silico approach to predicting the solubility and compatibility between substances [15])
CCC	Countercurrent Chromatography
GPC	Gel Permeation Chromatography
pTLC	Preparative Thin Layer Chromatography
pLC	Preparative Liquid Chromatography
GC × GC	Comprehensive Two-Dimensional GC
MS	Mass Spectrometry
MS/MS	Tandem Mass Spectrometry
NMR	Nuclear Magnetic Resonance
